# Propagule pressure does not consistently predict the outcomes of exotic bird introductions

**DOI:** 10.7717/peerj.7637

**Published:** 2019-09-11

**Authors:** Michael P. Moulton, Wendell P. Cropper

**Affiliations:** 1 Department of Wildlife Ecology and Conservation, University of Florida, Gainesville, FL, USA; 2 School of Forest Resources and Conservation, University of Florida, Gainesville, FL, USA

**Keywords:** Bird introductions, Propagule pressure, Anna Karenina

## Abstract

Some have argued that the role of propagule pressure in explaining the outcomes of bird introductions is well-supported by the historical record. Here, we show that the data from a large published database (including 832 records with propagule information) do not support the conclusion that propagule pressure is the primary determinant of introduction success in birds. A few compendia of historical reports have been widely used to evaluate introduction success, typically by combining data from numerous species and introduction locations. Very few taxa, other than birds, have usable spatially explicit records of introductions over time. This availability of data inflates the perceived importance of bird analyses for addressing factors related to invasion success. The available data allow limited testing of taxonomic and site-level factors of introduction outcomes. We did find significant differences in effort and success probabilities among avian orders and across highly aggregated spatial regions. As a test of a standard and logical expectation of the propagule pressure hypothesis, we concentrated on introductions with the smallest propagules, because it is for these the hypothesis is most likely to be correct. We analyzed the effect of numbers released in small propagules (two through 10) for 227 releases. Weighted linear regression indicated no significant effect of propagule size for this range of release size. In fact, the mean success rate of 28% for propagules of 2–10 isn’t significantly different than that of 34% for propagules of 11–100. Following the example of previous analyses, we expanded the statistical test of propagule pressure to include the full range of release numbers. No significant support for the propagule pressure hypothesis was found using logistic regression with either logit or complementary log-log link functions.

## Introduction

Successful species introductions are all alike in that every obstacle to their establishment must have been overcome: Unsuccessful introductions can each be unsuccessful for a different reason. This notion is an extension of the observation by Leo Tolstoy in his novel (Anna Karenina) describing happy vs. unhappy families. It has been extended previously to include the field of ecological risk assessments (Moore, 2001). Here, we suggest that the idea also should be applied to the dynamics of species’ introductions.

Duncan, Blackburn & Sol (2003), Blackburn, Lockwood & Cassey (2009a) and Blackburn et al. (2011a) noted that the outcomes of species’ introductions could be influenced by characteristics of the species introduced, the environment where the introduction occurs, or various aspects of the specific releases. We agree that introductions could fail for reasons in any one of these categories. However, we disagree with those who have focused on propagule pressure, meaning the total number of individuals released, as the strongest predictor of introduction outcomes. Moreover, simply emphasizing the importance of population size to introduction outcomes can mistakenly overshadow the importance of species-level (Blackburn, Cassey & Lockwood, 2009b; Cassey, Prowse & Blackburn, 2014) and location-level factors (Moulton, Cropper & Avery, 2011, 2012b, 2013; Moulton et al., 2012a; Moulton & Cropper, 2014a, 2014b, 2015), as well as other human related, event-level factors.

Propagule pressure is but one reason introductions might fail and its primacy as an explanation has been greatly inflated. First, we present the arguments for the perceived primacy. Second, we critique the assumptions and third, the data presented to support their primacy. Fourth, we present evidence that strongly supports other explanations for why introductions fail. Then, in a new analysis, we consider a large published database that includes 832 records with propagule information. These data do not support the conclusion that propagule pressure is the primary determinant of introduction success in birds.

### Primacy arguments

Veltman, Nee & Crawley (1996) and Green (1997) in studies of introduced birds to New Zealand reported that number of individuals released was the principal factor in influencing introduction fate. Duncan (1997), in a study limited to passeriform birds introduced to New Zealand, concluded that striking patterns in invasion success could be simply the result of variation in introduction effort. Cassey et al. (2004) expanded this to a “global” level and also drew the conclusion that introduction effort (as gauged by propagule pressure) was the “strongest correlate of introduction success.” Lockwood, Cassey & Blackburn (2005) proposed that propagule pressure was the “key element” in determining whether introductions succeed or fail. Vall-llosera & Sol (2009) stated that number of individuals released (i.e., propagule pressure) was, “… the main factor influencing the establishment success of introduced animals…”. This was a recurrent theme in Blackburn, Lockwood & Cassey (2009a) and in Blackburn et al. (2011b, 2013).

These studies all relied on compilations of numbers of individuals released per species taken from historical records. There are two major problems with this approach. The first is the implicit assumption that individuals of species were released principally, or exclusively, for establishing the species and that releases were discontinued once the species was perceived to be “established.” The second implicit assumption is that for unsuccessful introductions the number released was either insufficient or not the cause of the failure. It must also be true that for successful introductions the actual number of individuals or introduction events needed is confounded by introductions into established populations.

The popularity of this perspective has advanced despite numerous studies that show equally important roles for location-level factors (Moulton & Cropper, 2014a; Moulton et al., 2018; Redding et al., 2019) and species-level factors (Blackburn, Cassey & Lockwood, 2009b; Sol et al., 2005, 2012; Cassey, Prowse & Blackburn, 2014).

Some, for example Blackburn, Lockwood & Cassey (2015a) and Blackburn et al. (2015b), have likened the well-known relationship between small populations and increased chances for extinction in natural populations to small propagules of introduced species. However, at any arbitrarily small size, natural populations that are declining are very different from introduced populations that are increasing. Indeed, Cassey, Prowse & Blackburn (2014) noted that in a model analysis of introduced species dynamics, net reproductive rate (*R*_0_) was consistently more important than propagule pressure in determining introduction outcomes across three species-life-history types. The propagule pressure hypothesis involves too many assumptions to rigorously test or support it as the primary factor.

It is also clear that the data involved are too limited and contain too many inaccuracies and inconsistencies for such a conclusion (Moulton et al., 2010, 2012a, 2014, 2018; Moulton, Cropper & Avery, 2011, 2012b, 2013; Moulton & Cropper, 2014a, 2015, 2016). Simple modeling of numerical responses thus assumes that the habitat in the new location was suitable, that impacts of predators or competitors are inconsequential, that the demographic factors of the individuals released did not predispose them to have abnormally reduced population growth rates (e.g., too many males not enough females, the individuals were not too old or too inbred) and that a host of potentially negative human-related factors (e.g., time in captivity or in transit, etc.) were overcome.

Accumulating many examples of inappropriate tests (aggregating over species, space, and time) does not increase the value of the comparisons. Historical records that are typically used for these analyses commonly list just a sum of individuals released. In using these values, one must ignore the fact that birds often are released for reasons other than simple establishment. Indeed, some species have been introduced multiple times to a location in order to control insect pests, increase local biodiversity, provide game for hunters, and perhaps other human motivations (Blackburn, Lockwood & Cassey, 2009a). The numbers ultimately released also may simply reflect the costs of transport or captive breeding, and thus are a poor indicator of the actual number of individuals needed for establishment. Similarly, large numbers of birds, particularly game birds (i.e., Galliformes) sometimes have been introduced into already established populations simply to increase the numbers of a desired species to satisfy hunters (McDowall, 1994; Blackburn, Lockwood & Cassey, 2009a). Thus, correlations between numbers released and apparent successes, conflates the actual number necessary to ensure success and the number introduced after establishment of a viable population.

In one of the few studies that examines several factors together—Moulton et al. (2018) found that 76% of game bird species (13/17) always failed when released in the continental USA, suggesting they were ill suited as species (or perhaps in the place introduced), 77% (10/13) of the species that always failed in the locations to which they were introduced in the USA were successful somewhere else in the world.

### Looking for propagule pressure where it is most likely

It would not be surprising if instances where introduction failure was due chiefly, or even exclusively, to inadequate propagule pressure exist. But, as noted by Sol et al. (2012) and Redding et al. (2019), it is logical to assume that this would most likely be observed when propagules are very small. At the other end of the spectrum, propagule pressure has been questioned as an explanation of why releases of large numbers of individuals (e.g., >100; per Blackburn, Lockwood & Cassey, 2009a) of a species are sometimes unsuccessful, or why sometimes very small numbers of individuals (i.e., <10) released are successful.

In this study, we re-examine a large database used in an analysis of introduced birds (Sol et al., 2012). These data represent a valuable contribution but are severely limited by spatial and temporal aggregation. Thus, with regard to spatial aggregation, Sol et al. (2012) treated the Hawaiian Islands and the USA as single locations. The land area of the conterminous 48 states is in excess of 8,000,000 km^2^, whereas the entire Hawaiian archipelago encompasses just 16,663 km^2^. We know from previous studies (Moulton et al., 2018) that species that succeeded in some parts of the USA failed in other parts. Of course, any possible environmental differences across the USA, that could influence introduction outcomes, would vanish when subjected to this level of aggregation.

With respect to temporal aggregation, the total number of individuals released as reported in this dataset gives no indication of the time span over which the releases occurred. Thus, for a sum of 100 individuals released we cannot determine if this was many releases of a few individuals over many years or fewer releases of many individuals over a shorter time span. The problem expands as the sums increase: the 12,004 Chilean Tinamous (*Nothoprocta perdicaria*) listed by Banks (1981) or the 4,114,235 Common Pheasants (*Phasianus colchicus*) listed by Sol et al. (2012).

We show that significant differences exist in the probability of success for species in different orders, and across different regions. We show that propagule pressure fails to predict many introduction outcomes, reinforcing the conclusion that species-level or location-level factors in some cases are equally or more important than propagule pressure. Further, we show that differences in introduction success rates across taxonomic orders and locations exist and we argue that these could be of equal importance.

## Methods and materials

We began by log-transforming the 832 records with propagule information listed by Sol et al. (2012). In our first analysis we used a weighted regression model of the probability of introduction success against propagule sizes for the smallest propagules (2–10 individuals). We then extended this analysis by calculating the probability of success in propagule size categories with spans of 10 or more releases (we used larger categories at the high end of the scale). We then attempted to fit a logistic regression model of probability of success against log-transformed propagule size categories.

We adopted this procedure because of the substantial uncertainties in propagule sizes per release (Moulton, Cropper & Broz, 2015). Additionally, previous analyses that used extremely large releases compiled over many species, over long spans of time and spatial scale inflate the number of degrees of freedom leading to spurious results.

To assess species-level differences, we compared the success rates for species in 12 orders and across locations (We included three releases from the order Craciformes with the Galliformes). We used a simple analysis of variance to compare probabilities of success to the log-transformed propagule sizes across the different orders listed by Sol et al. (2012). We further used orthogonal contrasts to compare release sizes of species in the Galliformes with those of the Passeriformes, and also the release sizes of Galliformes with the combination of the four other best represented orders: Anseriformes; Passeriformes; Columbiformes and Psittaciformes.

The Sol et al. (2012) data are too crude for a detailed assessment of the importance of location-level variables. However, the bulk of the releases (659 of 832) occurred in just four regions: Australia (97); Hawaii (68); New Zealand (231); USA (263). We compared success rates across locations with a test of independence.

For all our univariate, linear regression and logistic regression analyses we used procedures in SAS Institute Inc. (2009).

## Results

**Figure 1:**
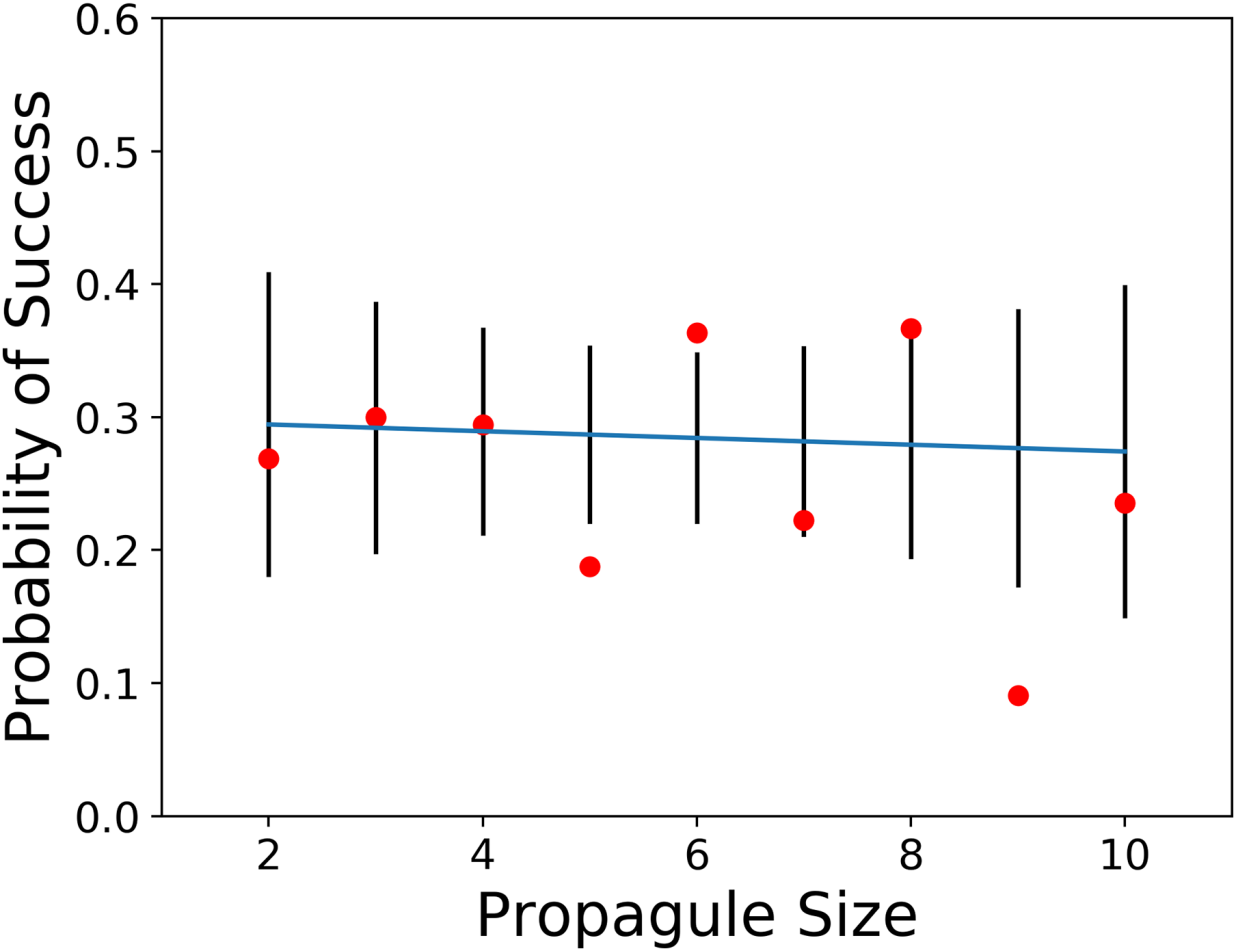
Plot of probability of introduction success vs. propagule sizes of two through 10 with observed values, and 95% confidence limits (data from Sol et al., 2012).

The first prediction of the propagule pressure hypothesis is that introductions with the smallest propagule sizes should mostly fail, whereas the releases in groups with larger propagule sizes should mostly succeed. To test this idea, we used a weighted regression (weighted by the number of observations used to calculate probability of success) of the probability of successful introduction against propagule sizes of two through 10 individuals (see Table 1). Over the nine release categories the total number of observations ranged from nine to 67. The result of this analysis was no significant relationship between probability of success and propagule size (*F* = 0.06, *p* > *F* = 0.82). The release of between 2 and 10 individuals (see Fig. 1) comprised 227 of the 832 releases (27.3%) with propagule information in Sol et al. (2012).

**Table 1:** Introduction fates for small propagule sizes.

Number released	Unsuccessful	Successful	p (S)
2	49	18	0.269
3	7	3	0.3
4	24	10	0.294
5	13	3	0.188
6	21	12	0.364
7	7	2	0.222
8	19	11	0.367
9	10	1	0.091
10	13	4	0.235

**Note:**

Successful (Suc) and Unsuccessful (Uns) releases of 2–10 and probabilities of success (p (S) = Successful/Total) per release number.

**Figure 2:**
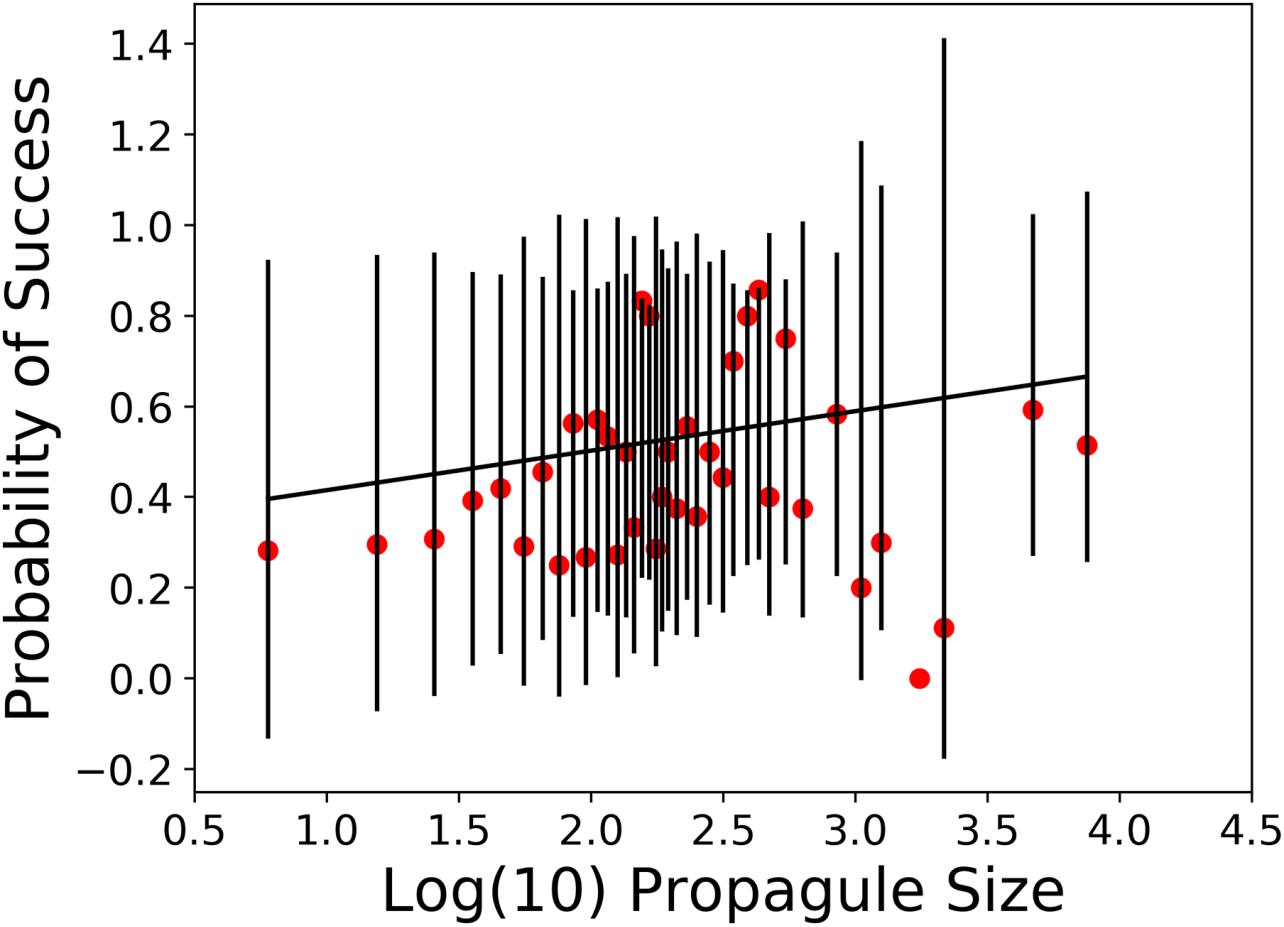
Weighted general linear model of probability of introduction success vs. log propagule size class. With 95% confidence limits about individual observations. (data from Sol et al., 2012).

The second prediction of propagule pressure is that as the number of individuals released increases, the chances for establishment success also increase but only up to a point. We argue that previous analyses of propagule pressure effects on introduction outcome using logistic regression suffer from the degree of aggregation in the releases. In the Sol et al. (2012) data set, 89 of the 202 species (44%) were released a single time and five species of Galliform birds made up 280 releases (34%). Based on this, we calculated probabilities of introduction success in 38 different propagule size classes (Table 2). A plot of these probability values is shown in Fig. 2. Our dependent variable (probability of success) followed a Gaussian distribution and we specified both logit and complementary log-log link functions (Duncan et al., 2014). In this analysis, we found the probability of success was not significantly related to log propagule size regardless of which link function we used (see Table 3).

**Table 2:** Probabilities of success for 832 releases.

Mid prop cat	Log mid prop cat	Successful	Unsuccessful	p (S)
6	0.77815125	64	163	0.282
15.5	1.1903317	32	76	0.296
25.5	1.40654018	16	36	0.308
35.5	1.55022835	11	17	0.393
45.5	1.6580114	13	18	0.419
55.5	1.74429298	7	17	0.292
65.5	1.8162413	10	12	0.455
75.5	1.87794695	3	9	0.25
85.5	1.93196611	9	7	0.563
95.5	1.98000337	4	11	0.267
105.5	2.02325246	8	6	0.571
115.5	2.06258198	8	7	0.533
125.5	2.09864373	3	8	0.273
135.5	2.1319393	2	2	0.5
145.5	2.16286299	2	4	0.333
155.5	2.19173039	5	1	0.833
165.5	2.218798	4	1	0.8
175.5	2.24427712	2	5	0.286
185.5	2.26834391	2	3	0.4
195.5	2.29114676	9	9	0.5
210.5	2.3232521	3	5	0.375
230.5	2.36267093	5	4	0.556
250.5	2.39880773	5	9	0.357
280.5	2.44793287	3	3	0.5
315	2.49831055	4	5	0.444
345.5	2.53844805	7	3	0.7
390.5	2.59162104	4	1	0.8
430.5	2.63397316	6	1	0.857
470.5	2.67255963	6	9	0.4
545.5	2.73679475	6	2	0.75
630.5	2.79968509	3	5	0.375
850.5	2.92967432	7	5	0.583
1,050.5	3.02139606	1	4	0.2
1,250.5	3.0970837	3	7	0.3
1,745.5	3.24191985	0	11	0
2,160.5	3.33455427	1	8	0.111
4,700.5	3.67214406	16	11	0.593
7,501	3.87511917	17	16	0.515

**Note:**

Raw numbers for p (S) plot. Mid prop cat, the midpoint of the propagule size categories; log mid prop cat, common logarithms of the midpoint of each propagule category as a measure of propagule size; Successful, Unsuccessful, number of successful and unsuccessful releases per category, respectively; p (S) calculated as in Table 1.

**Table 3:** Results of logistic regression of probability of successful establishment vs. log propagule size categories with logit and complementary log-log link functions.

Logit link function					
Effect	Estimate	S.E.	d*f*	*t*-value	*P* > |*t*|
Intercept	−0.4663	0.4994	36	−0.93	0.36
Log prop cat	0.1078	0.2058	36	0.52	0.60
Scale	0.0164	0.004			

Complementary log-log link function					
Effect	Estimate	S.E.	d*f*	*t*-value	*P* > |*t*|
Intercept	−0.7040	0.3684	36	−1.91	0.06
Log prop cat	0.0765	0.1504	36	0.51	0.61
Scale	0.0410	0.01			

Although we have serious reservations about doing this analysis on individual releases for such a diverse set of species, locations and times, it has been a common approach in addressing this problem. With this caveat in mind, a single logistic regression on the individual releases yielded a significant positive slope estimate (d*f* = 830). However, this is not compelling support for propagule pressure as a dominant predictor of introduction outcome. An analysis based on unfounded assumptions cannot be considered valid even if a statistically significant relationship emerges. Moreover, given the large number of degrees of freedom in this model, statistical significance could easily lead to improper interpretations.

### Taxonomic differences

We have inferred from prior analyses (Moulton et al., 2018) that significant differences exist across taxonomic groups. The data here are too limited for proper tests of many species level effects, again due to the above mentioned aggregation problems. Sol et al. (2012) identified demographic features associated with introduction fates across 202 species with propagule information. To address potential taxonomic differences, we compared probability of success among taxonomic orders. Most of the orders in Sol et al. (2012) were represented by 10 or fewer releases (Table 4), the exception being the Ciconiiformes with 24 releases. However, Dickinson & Remsen (2013), in an updated taxonomy, treated species in these 24 releases as belonging to five different orders and did not consider the Ciconiiformes to be a valid order. Dickinson & Remsen (2013) also merged the three releases from the Craciformes of Sol et al. (2012) with the Galliformes. The majority of the 832 releases listed by Sol et al. (2012) belong to just five orders: Anseriformes (71); Columbiformes (31); Galliformes (407); Passeriformes (253) and Psittaciformes (17).

**Table 4:** Distribution of releases across 12 avian orders.

Orders	Releases	Species	Releases/species
Struthioniformes	5	3	1.7
Tinamiformes	10	7	1.4
**Anseriformes**	71	19	3.7
Galliformes*	407	53	7.7
Apodiformes	1	1	1
Gruiformes	1	1	1
**Columbiformes**	31	15	2.1
Ciconiiformes**	24	9	2.7
Strigiformes	9	3	3
Coraciiformes	3	1	3
**Psittaciformes**	17	14	1.2
**Passeriformes**	253	76	3.3

**Notes:**

* Includes the Craciformes.

** Now considered to be five separate orders.

Bold font orders represent more than 93% of the releases.

Although as mentioned previously we cannot effectively test species-level effects within these data due to aggregation problems, we can conduct tests at higher taxonomic levels. With this in mind, we compared log numbers per release across the five best represented orders. In an ANOVA the five best represented orders differed significantly.

**Figure 3:**
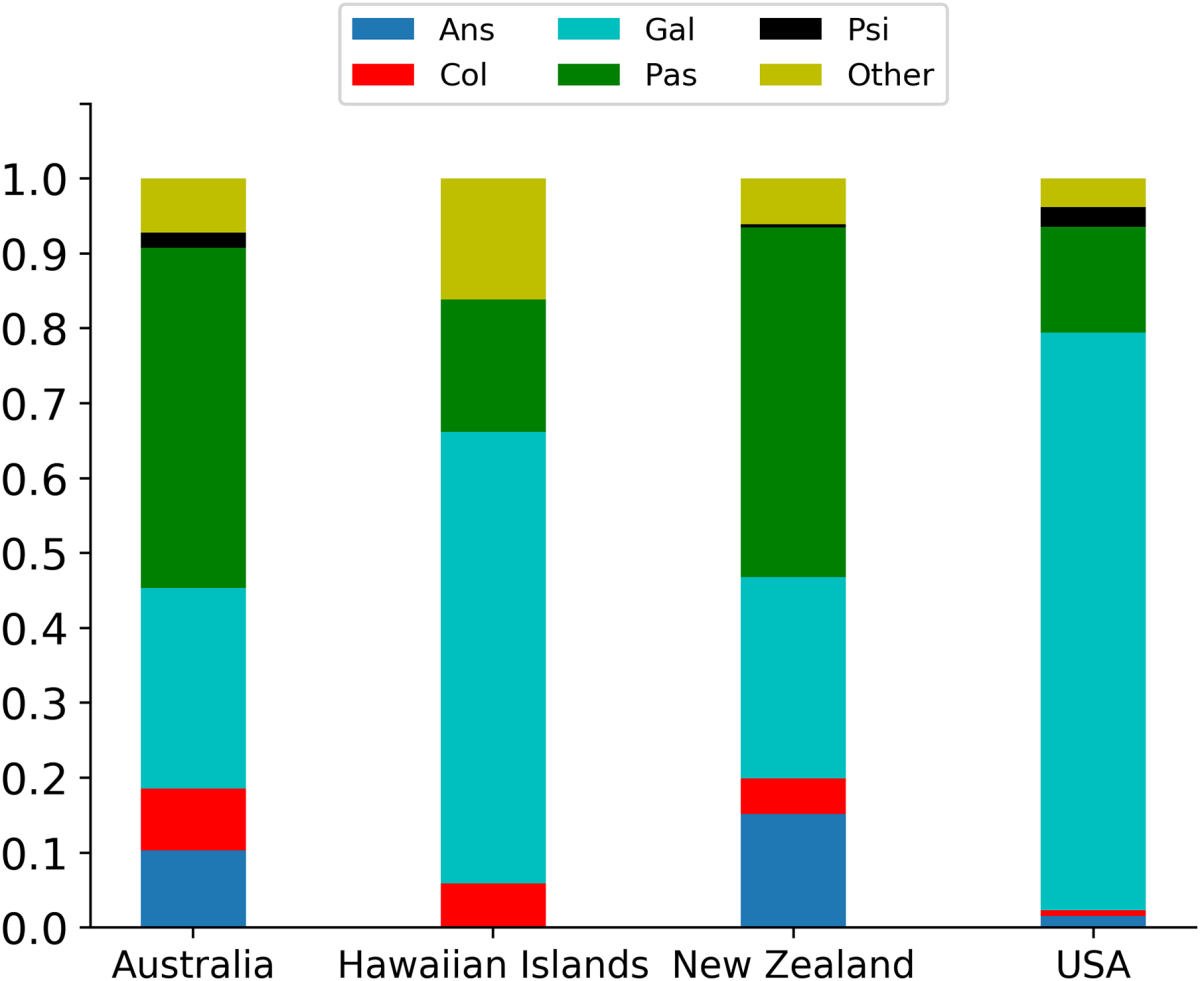
Percentage breakdown of the five principally involved taxonomic orders: Ans, Anseriformes; Col, Columbiformes; Gal, Galliformes; Pas, Passeriformes; Psi, Psittaciformes; Other, multiple orders) in the four locations with the most releases (Australia).

The spatial scale of the data also does not allow for a detailed analysis of location-level factors. However, of the 46 locations listed by Sol et al. (2012), 38 (82%) had fewer than 10 releases with the bulk of the releases (659/832 = 79%) in just four locations: Australia (97); Hawaiian Islands (68); New Zealand (231); and USA (263). The introduction success rates varied across these sites (see Table 5), with the greatest success rate in the Hawaiian Islands and the lowest in the USA. One might reasonably conclude that this could be the result of asymmetrical taxonomic distribution of releases to these sites, and indeed this was the case. Thus, for Australia and New Zealand the releases involved species of the Passeriformes, whereas in the Hawaiian Islands and the USA most were from the Galliformes (Fig. 3).

**Table 5:** Taxonomic breakdown of releases by most prominent orders and locations.

Location	Ans	Col	Gal	Pas	Psi	Oth	U	S	p (S)
Australia	10	8	26	44	2	7	62	35	0.36
Hawaiian Islands	0	4	41	12	0	11	32	36	0.53
New Zealand	35	11	62	108	1	14	138	93	0.40
USA	4	2	203	37	7	10	189	74	0.28

**Note:**

Legend: Ans, Anseriformes; Col, Columbiformes; Gal, Galliformes; Pas, Passeriformes; Psi, Psittaciformes; Oth, various orders. Number of unsuccessful (U) and successful (S) releases and probabilities of success (p(S)) for four locations.

## Discussion

Proponents of the propagule pressure hypothesis argue that introductions fail mostly because insufficient numbers of individuals were released (Lockwood, Cassey & Blackburn, 2005; Blackburn, Lockwood & Cassey, 2009a). They further argue that increasing propagule size leads to correspondingly higher chances for establishment success up to some approximately estimated point. In a recent study, Redding et al. (2019) used a Bayesian hierarchical regression analysis to evaluate factors associated with introduction outcomes for a wide range of bird species and locations. This analysis concluded that introduction location, and the presence of other non-native species, are the primary determinants of successful establishment. Redding et al. (2019) additionally suggested that propagule pressure was an important secondary factor for predicting introduction outcomes. Several problems with this analysis could have inflated the modeled strength of propagule pressure. Redding et al. (2019) aggregated multiple introduction events into a single number for cumulative propagule pressure with the implicit assumption that all were needed for establishment. Moreover, the data used on this analysis included a large number of Galliform birds (20.2% of 1,530 records). Managers often introduced these birds into previously established populations (Banks, 1981; Moulton, Cropper & Broz, 2015, 2018; Moulton & Cropper, 2014b).

When we regressed probabilities of success across propagule size categories, which included all 832 releases with propagule information, we found no evidence for increased probability of success with increased numbers released. We also did not detect a significant relationship between probability of success and propagule size for propagules of 2 through 10 individuals. The greatest response to propagule size is expected at very low numbers. In fact, 19 of the 67 introductions of sizes two through 10 generated a mean probability of success of 28%, which did not differ significantly from the mean value for releases of 11–100 (105/308 = 34%).

In our re-examination of the Sol et al. (2012) data, significant differences occurred in numbers of individuals released for species in the five taxonomic orders with 10 or more releases. However, the variation in levels of introduction effort did not lead to correspondingly higher probabilities of success in these orders. Thus, Galliform species although released in significantly higher numbers than species from other orders, had the lowest success rate. And this can be attributed to aggregation problems in the data.

Significant differences also occurred across introduction sites. The number of releases per location declined steeply after the four sites with the greatest numbers of releases: Australia; Hawaiian Islands; New Zealand; USA. Location-level variations were not the focus of Sol et al. (2012) and thus the “sites” listed are highly variable in area and likely in environmental heterogeneity as well. As an example of this, Moulton et al. (2018) showed that releases of Common Pheasants in the USA invariably failed in nine southeastern states regardless of the release sizes. Similarly, Chukars, despite released in very large numbers (e.g., 85,000 in Minnesota (Christensen, 1970)) failed to become established in any of the eastern states. Nevertheless, of the four main locations, the Hawaiian Islands, the smallest land area, and arguably the lowest environmental heterogeneity, had the highest introduction success rates. In contrast, the USA, which we infer to mean the lower 48 states, had the greatest land area of the four sites and simultaneously the lowest introduction success rate. It is logical to infer that this difference in success rates was the product of different species having been released in these different locations. However, the Hawaiian Islands and the USA both shared a proportionally higher numbers of Galliform introductions, whereas Australia and New Zealand had higher numbers of Passeriform species. Moreover, (Moulton & Cropper, 2014a) showed that for a set of Passeriform species introduced to New Zealand, Australia and the USA had the lowest success rates in the USA, suggesting a predominant role for environmental characteristics. Based on our analyses in this, and other studies, we suggest that the Anna Karenina principle (multiple reasons for introduction failure) better reflects establishment outcomes than a fixation on the total number released.

## Conclusions

Historical records of bird introductions have often been used to assess ecological and environmental factors associated with the probability of successful establishment. Although propagule pressure has often been advanced as a primary predictor of introduction outcome, we found no support for this hypothesis in a global data set with 832 records with propagule information. For the smallest propagules (2–10 individuals), where the strongest impact should be visible, we found no significant effect of propagule size. Similarly, across the full release range of propagule sizes we found no significant support for propagule pressure.

## Supplemental Information

10.7717/peerj.7637/supp-1 Supplemental Information 1 Individual releases for 202 species. Numbers of individuals released per species arranged in groups.

## Additional Information and Declarations

### Data Availability

The following information was supplied regarding data availability: Raw data for all our analyses are available as a Supplemental File.
